# Supplementary material for: Artificial Diets Modulate Infection Rates by Nosema ceranae in Bumblebees
Source: Microorganisms. 2021 Jan 12;9(1):158. doi: 10.3390/microorganisms9010158 (PMC7827189; doi:10.3390/microorganisms9010158)
Supplement: Supplementary file 1 [file microorganisms-09-00158-s001.pdf]

Supplementary materials

Table S1. Number of bumblebees positive to *N. ceranae* in a PCR and showing spores at different days post exposure (dpe) per spore dosages and diet treatment. LP: low-protein diet; HP: high-protein diet.

		150K			300K		
**Diet**	**Dpe**	**Dead**	**PCR positive**	**Spores**	**Dead**	**PCR positive**	**Spores**
LP	1	0	0	0	0	0	0
LP	2	3	3	0	2	2	0
LP	3	5	2	1	3	2	1
LP	4	5	3	1	7	6	1
LP	5	4	0	0	7	1	0
LP	6	6	3	1	4	2	2
LP	7	2	1	0	3	2	1
LP	8	3	1	0	4	2	0
LP	9	3	1	0	0	0	0
LP	10	0	0	0	2	2	0
LP	11	1	1	0	0	0	0
LP	12	0	0	0	0	0	0
LP	13	0	0	0	0	0	0
LP	14	1	0	0	0	0	0
LP	15	1	1	0	1	1	0
LP	16	0	0	0	1	1	0
LP	17	0	0	0	1	0	0
LP	18	0	0	0	1	1	0
LP	19	2	1	0	0	0	0
LP	20	6	6	4	9	9	4
LP	**TOTAL**	***42***	***23***	***7***	***45***	***31***	***9***
HP	1	0	0	0	0	0	0
HP	2	2	1	1	2	1	0
HP	3	4	3	0	3	3	3
HP	4	2	1	0	4	2	0
HP	5	4	0	0	5	4	1
HP	6	5	2	2	3	1	0
HP	7	5	0	0	3	1	1
HP	8	13	6	1	9	1	0
HP	9	4	0	0	3	0	1
HP	10	3	0	0	6	2	0

HP	11	2	0	0	1	0	0
HP	12	3	1	0	0	0	0
HP	13	0	0	0	0	0	0
HP	14	0	0	0	0	0	0
HP	15	0	0	0	1	0	0
HP	16	0	0	0	0	0	0
HP	17	0	0	0	0	0	0
HP	18	0	0	0	0	0	0
HP	19	0	0	0	0	0	0
HP	20	0	0	0	1	0	0
HP	**TOTAL**	***47***	***14***	***4***	***41***	***15***	***6***

Table S2. Tukey post-hoc test to account for pairwise comparisons between bumblebees in different diets and spore treatments. Groups are defined by diet.dosage.infection. Diet: C (low-protein), P (high-protein). Dosage: 0, 150, 300. Infection: 0 (not-infected), 1 (infected).

Group 1	Group2	Estimate	Std. Error	z-value	pr(>|z|)	
C.150.0	C.0.0	-1.10587	0.14964	-7.39	<0.01	***
C.150.0	P.0.0	-0.07837	0.12572	-0.623	1	
C.150.1	C.0.0	-0.4991	0.15674	-3.184	0.0554	.
C.150.1	P.0.0	0.5284	0.13485	3.918	<0.01	**
C.150.1	C.150.0	0.60677	0.15356	3.951	<0.01	**
C.150.1	P.150.0	0.59391	0.14048	4.228	<0.01	**
C.150.1	C.300.0	0.69054	0.16983	4.066	<0.01	**
C.150.1	P.300.0	0.44123	0.14369	3.071	0.0774	.
C.300.0	C.0.0	-1.18964	0.16624	-7.156	<0.01	***
C.300.0	P.0.0	-0.16214	0.14513	-1.117	0.9927	
C.300.0	C.150.0	-0.08377	0.16228	-0.516	1	
C.300.0	P.150.0	-0.09663	0.15034	-0.643	1	
C.300.1	C.0.0	-0.44593	0.14664	-3.041	0.0845	.
C.300.1	P.0.0	0.58157	0.12332	4.716	<0.01	***
C.300.1	C.150.0	0.65995	0.14374	4.591	<0.01	***
C.300.1	P.150.0	0.64709	0.12947	4.998	<0.01	***
C.300.1	C.300.0	0.74372	0.161	4.619	<0.01	***
C.300.1	P.300.0	0.49441	0.13298	3.718	<0.01	**
C.300.1	C.150.1	0.05318	0.1519	0.35	1	
C.300.1	P.150.1	0.79892	0.16081	4.968	<0.01	***
P.0.0	C.0.0	-1.0275	0.12842	-8.001	<0.01	***
P.150.0	C.0.0	-1.09301	0.13448	-8.127	<0.01	***
P.150.0	P.0.0	-0.06551	0.10885	-0.602	1	
P.150.0	C.150.0	0.01286	0.13169	0.098	1	
P.150.1	C.0.0	-1.24485	0.16532	-7.53	<0.01	***
P.150.1	P.0.0	-0.21735	0.1448	-1.501	0.9308	
P.150.1	C.150.0	-0.13897	0.16241	-0.856	0.9993	
P.150.1	P.150.0	-0.15183	0.15006	-1.012	0.9969	
P.150.1	C.300.0	-0.0552	0.17787	-0.31	1	

P.150.1	P.300.0	-0.30451	0.15307	-1.989	0.6703	
P.150.1	C.150.1	-0.74574	0.16975	-4.393	<0.01	***
P.300.0	C.0.0	-0.94034	0.13815	-6.807	<0.01	***
P.300.0	P.0.0	0.08716	0.11305	0.771	0.9998	
P.300.0	C.150.0	0.16554	0.13502	1.226	0.9843	
P.300.0	P.150.0	0.15268	0.11973	1.275	0.9787	
P.300.0	C.300.0	0.24931	0.15327	1.627	0.8847	
P.300.1	C.0.0	-1.35445	0.15933	-8.501	<0.01	***
P.300.1	P.0.0	-0.32695	0.13784	-2.372	0.392	
P.300.1	C.150.0	-0.24858	0.15619	-1.592	0.8995	
P.300.1	P.150.0	-0.26144	0.14335	-1.824	0.7816	
P.300.1	C.300.0	-0.16481	0.17221	-0.957	0.9981	
P.300.1	P.300.0	-0.41411	0.14649	-2.827	0.1492	
P.300.1	C.150.1	-0.85535	0.16382	-5.221	<0.01	***
P.300.1	P.150.1	-0.1096	0.17212	-0.637	1	
P.300.1	C.300.1	-0.90852	0.15456	-5.878	<0.01	***
